# Transporter associated with antigen processing 1 (TAP1) expression and prognostic analysis in breast, lung, liver, and ovarian cancer

**DOI:** 10.1007/s00109-021-02088-w

**Published:** 2021-05-28

**Authors:** Anika Tabassum, Md. Nazmus Samdani, Tarak Chandra Dhali, Rahat Alam, Foysal Ahammad, Abdus Samad, Tomasz M. Karpiński

**Affiliations:** 1 Biochemistry Department, School of Life Sciences, Independent University, Dhaka, 1229 Bangladesh; 2 Department of Pharmacy, University of Dhaka, Dhaka, 1000 Bangladesh; 3 Department of Biotechnology and Genetic Engineering, Khulna University, Khulna, 9208 Bangladesh; 4 Laboratory of Computational Biology, Biological Solution Centre (BioSol Centre), Jashore, 7408 Bangladesh; 5 Department of Genetic Engineering and Biotechnology, Faculty of Biological Science and Technology, Jashore University of Science and Technology, Jashore, 7408 Bangladesh; 6 Department of Biological Sciences, Faculty of Science, King Abdulaziz University (KAU), Jeddah, 21589 Saudi Arabia; 7 Chair and Department of Medical Microbiology, Poznań University of Medical Sciences, Wieniawskiego 3, 61-712 Poznań, Poland

**Keywords:** TAP1, Transcriptional expression, Methylation analysis, Survival analysis, Co-expression, Pathway analysis, Health informatics

## Abstract

**Abstract:**

Transporter associated with antigen processing 1 (TAP1) is a transporter protein that represent tumor antigen in the MHC I or HLA complex. Any defect in the TAP1 gene resulting in inadequate tumor tracking. TAP1 influences multidrug resistance (MDR) in human cancer cell lines and hinders the treatment during chemotherapeutic. The association of TAP1 in cancer progression remains mostly unknown and further study of the gene in relation with cancer need to conduct. Thus, the study has designed to analyze the association between the TAP1 with cancer by computationally. The expression pattern of the gene has determined by using ONCOMINE, GENT2, and GEPIA2 online platforms. The protein level of TAP1 was examined by the help of Human Protein Atlas. Samples with different clinical outcomes were investigated to evaluate the expression and promoter methylation in cancer vs. normal tissues by using UALCAN server. The copy number alteration, mutation frequency, and expression level of the gene in different cancer were analyzed by using cBioPortal server. The PrognoScan and KM plotter platforms were used to perform the survival analysis and represented graphically. Additionally, pathway and gene ontology (GO) features correlated to the TAP1 gene were analyzed and presented by bar charts. After arranging the data in a single panel like correlating expression to prognosis, mutational and alterations characteristic, and pathways analysis, we observed some interesting insights that emphasized the importance of the gene in cancer progression. The study found the relationship between the TAP1 expression pattern and prognosis in different cancer tissues and shows how TAP1 affects the clinical characteristics. The analytical data presented in the study is vital to learn about the effect of TAP1 in tumor tissue, where previously studies showing contradicting expression of TAP1 in cancer tissue. The analyzed data can also be utilized further to evade the threats against chemotherapy. Overall, the study provided a new aspect to consider the role of TAP1 gene in cancer progression and survival status.

**Key messages:**

• This study demonstrated, for the first time, a correlation between the TAP1 gene and tumor progression.

• An upregulation of TAP1 mRNA was demonstrated in various cancer types.

• This study reported a significant negative correlation for TAP1 gene expression and the survival rate in different cancer types.

**Supplementary Information:**

The online version contains supplementary material available at 10.1007/s00109-021-02088-w.

## Introduction

Cancer has become a major contributor to mortality worldwide. In 2018, the global cancer death rate was estimated to be about 9.6 million [1], and in 2020, 606,520 cases of deaths were estimated in the United States (US) alone [2]. At this condition, the significance of finding better treatments is very high, even though we have options like chemotherapy, immunotherapy, or radiotherapy, but have some limitations [3]. We tend to dive into the cellular mechanisms and immunology to understand tumor progression better. A great role has been featured by ATP-Binding Cassette (ABC) transporters in the development of cancer and, also in the immune response towards cancer [4].

The ABC superfamily regulates the passage for ions and substrates, through cellular and organelle membranes [4]. Transporter associated with antigen processing 1 (TAP1) protein resides in the ABCB (ATP binding cassette subfamily B member) subfamily [4]. It forms a heterodimer with TAP2 and helps to the transportation of cytosolic proteins into the endoplasmic reticulum. When TAP exports the antigenic peptides to the ER, the major histocompatibility complex I (MHC-I) are able to present the antigenic peptides on the surface of the host cell. These displayed peptides are then recognized by the T-lymphocytes and are inspected. The delivery during the antigen processing is important for detection of viral and tumor antigens and has seen to be hampered for the tumor to escape the detection [5–7]. It also has been reported that mutant TAP1 can have an effect in the MHC-I function of tumor surveillance [8]. A previous study proposed that TAP could be used as a cancer treatment via immunotherapy due to its importance in the peptide-MHC I complex and as it elevated the immune response [9].

Multidrug resistance (MDR) is one of the reasons of the failure in chemotherapy happened during metastasis and invasion [10]. Therefore, it is necessary to target novel molecules responsible for drug resistance in cancer cells and targeting the molecules will help to decrease the cancer related deaths. Different gene from the ABCB subfamily has found the MDR function [11], by which the cells confer simultaneous resistance to drugs in different forms and structures to the cancer cells [12]. ABC help to reduce the transportability of the cancer cell that limit the uptake ability of anti-cancer drug, increases the action of efflux pump, affects cell and organelle membranes [3], blocks cell death by anti-cancer drugs [13], detoxifies the drugs [14], and alters the cell cycle that nullifies the effect of the drugs on tumor cells [15]. It was discovered that TAP1 and P-glycoprotein domains, encoded by the ABCB1 gene, are homologically similar having same binding sites, and both are contribute to MDR [16]. The ABCB1 effluxes drugs/substrates generate from the cell membrane is resistant to anti-cancer drugs like anthracyclines, taxanes, and many other compounds [17].

While TAP1 upregulation has been linked to better targeting of tumor cells, there is contradictory evidence that shows the overexpression of TAP1 is associated in cancer development [18]. To know about the role of TAP1 in tumor development, further studies develop based on role of TAP1 in various cancers is require. A systematic computational analysis that utilizes most experimental data has not yet been reported. This type of studies is essential and will help us to determine the biomarker ability of the gene. Therefore, the study utilized various data from different databases and focuses on the expression pattern of TAP1 in various cancer, analyzed the gene for determine different clinical features, determined the copy number and amplification frequency to evaluate the role of gene in cancer. We also looked into the prognostic datasets and correlated genes to examine the prognosis and mechanisms related to TAP1. The reported analytical data will help us to understand more about the gene that can be implicated in further studies.

## Materials and methods

Differential expression of TCGA (The Cancer Genome Atlas) samples for different cancer was observed in comparison with normal complements through the ONCOMINE database (https://www.ONCOMINE.org/resource/login.html) [19–21]. The analysis of expression level was conducted considering a threshold parameter of; *p*-value: 1E-4, fold change: 2, gene ranking: 10%. The expression profile for genes throughout different cancer and their complementary normal tissues was detected through the GEPIA2 website (http://gepia2.cancer-pku.cn/) [22] and GENT2 online platform (http://gent2.appex.kr/gent2/) via HG-U133Plus2 database [23] with default parameters. The GTEx (Genotype-Tissue Expression) data and TCGA data were matched by the ANOVA differential method and were used to detect the expression with default threshold settings through the GEPIA2 website.

Expression of TAP1 protein in different cancer tissues and their counterpart normal tissue was retrieved as immunohistochemistry images retrieved Human Protein Atlas database (https://www.proteinatlas.org/) [24, 25].

Sample gene expression and promoter methylation analysis was conducted with the UALCAN website (http://ualcan.path.uab.edu/index.html) by comparing the TPM (transcript per million) and beta value, respectively [24, 26]. The TCGA and MET500 cohort were used in UALCAN to analyzed mRNA transcription count of cancer tissues with their normal counterparts, tissues in different cancer stages, different ethnicities and genders.

The number and location of the mutations in the peptide sequence were detected using cBioPortal (http://www.cbioportal.org/) [27, 28]. Frequency of alteration (Mutation, Amplification, Deep Deletion and Multiple Alterations) was investigated using the cBioPortal web. The tools of cBioPortal, OncoPrinter and MutationMaper, were used to visualize and analyze the RNA seq V2 profile sorted by cancer study [27, 28].

Transcriptional expression of TAP1 protein and its relation to patient survival in cancer patients was examined via PrognoScan and Kaplan–Meier plotter was utilized in examining the effect on overall survival (OS), relapse-free survival (RFS), and diseases-free survival (DFS) by multivariate and univariate investigation of TAP1 expression with *p*-value: 0.05 as a parameter [29].

The genomic investigation and visualization platform R2: Genomics Analysis and Visualization Platform (https://hgserver1.amc.nl/) was used to find the positively co-expressed genes of TAP1 of TCGA sample for breast, liver, lung, and ovarian cancer. A threshold of *p*< 0.01 and Bonferroni multiple comparison statistical test was considered for the investigation Venn diagrams (http://bioinformatics.psb.ugent.be/webtools/Venn/) was used to determine the common positively co-expressed genes of all the cancers. Enricher (http://amp.pharm.mssm.edu/Enrichr/enrich#) was used to present the pathways and ontologies sorted by p-value ranking as a bar diagram for the common positively co-expressed genes with default parameters [30]. All server details of our study in Table 1.

**Table 1:** List of the database and server utilize in the study

Database	Data type	Analysis type	Dataset	Website link
ONCOMINE	Gene expression	Cancer vs. normal analysis	1. Broad Tumorscape 2. Cell Line Panel 3. DNA Copy Number 4. Multi-Cancer Panel 5. Normal Tissue Panel 6. TCGA Datasets	(https://www.ONCOMINE.org/resource/login.html)
GEPIA2	Gene expression	Gene expression profile across all tumor samples and paired normal tissues	TCGA/GTEx dataset	(http://gepia2.cancer-pku.cn/)
GENT2	Gene expression	Tissue-wide gene expression profile across paired tissues	Public gene expression data sets	(http://gent2.appex.kr/gent2/)
UALCAN	Transcript per million	Transcript per million RNA molecule was compared across different categories	TCGA	(http://ualcan.path.uab.edu/index.html)
UALCAN	Promoter DNA methylation	Promoter DNA methylation was compared across different categories using beta value	TCGA	(http://ualcan.path.uab.edu/index.html)
cBioPortal	Genomic alteration	Frequency of mutation, amplification , deep deletion and multiple alterations across specific cancer was analyzed	TCGA	(http://www.cbioportal.org/)
	Mutation	Mutation across TAP1 protein was determined for specific cancer		
	Mutation and copy number alterations	Mutation and copy number alteration was identified in TAP1 mRNA expression for specific cancer		
The Human Protein Atlas	Protein expression	Protein expression data was compared across different categories using immunohistochemistry.	The tissue atlas	(http://www.proteinatlas.org/)
PrognoScan	Gene expression and patient prognosis	Gene expression was analyzed comparing patient prognosis such as overall survival (OS), disease-free survival (DFS) and relapse-free survival for cancer and normal patient	Public cancer microarray datasets	(http://dna00.bio.kyutech.ac.jp/PrognoScan/) (https://kmplot.com/analysis/)
R2: Genomics Analysis and Visualization Platform	Positively co-expressed genes	Identification and visualization of positively co-expressed genes of different cancers	TCGA	(https://hgserver1.amc.nl/) (http://bioinformatics.psb.ugent.be/webtools/Venn)
Enricher	Positively co-expressed genes	Signaling pathway and gene ontology analysis of positively co-expressed genes in KEGG human pathways 2019, Panther 2016, GO Biological Process, GO Molecular Function (2018) and GO Cellular Component ranking by *p*-value	TCGA	(http://amp.pharm.mssm.edu/Enrichr/enrich#)

## Results

### The TAP1 gene expression in different kinds of cancer

**Fig. 1:**
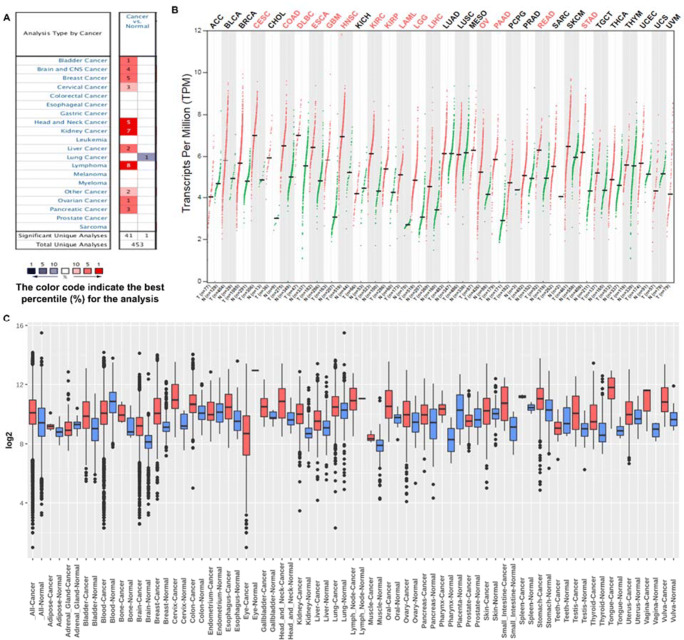
Expression of TAP1 in different cancer: **(A)** cancer vs. normal upregulation (red) and downregulation (blue) in left and right columns, respectively, with default parameters of a *p*-value: 1E-4, fold change: 2 and a % gene ranking: 10% for expression of mRNA in the ONCOMINE database. **(B)** mRNA transcription profiles for TAP1 in various cancer kinds were detected by TCGA database via GEPIA2 (Gene expression Profiling Interactive Analysis 2) website. In the dot plot, the red plot represents a tumor, and the blue plot represents normal tissues. Each dot represented the expression of samples. The tumor specimens were compared to their counterparts to observe the expression criteria. **(C)** TAP1 expression profile in different cancers and its counterparts were detected through Gene Expression across normal and tumor tissue (GENT2) with boxplot, where boxes with red color indicate cancer cells, boxes with blue color indicate normal cells, the middle line shows the median, and the dots are the outliers

To analyze the mRNA expression for the TAP1 gene in different kinds of cancer, we used three databases. We used ONCOMINE to look into the comparison of TAP1 mRNA expression for different cancer and their normal tissues. The total unique analysis was reported to be 453, among the 453 analysis 42 were significant (Fig. 1A). The comparison in ONCOMINE was done via Student’s *t* test, a *p*-value of 0.0001, and a fold change of 2. The mRNA levels were overexpressed in the bladder, brain and central nervous system (CNS), breast, cervical, head and neck, kidney, liver, lymphoma, ovarian, and pancreatic cancers while under-expressed only in the lung cancer, unlike the healthy tissues. The GEPIA2 server was then used to study the profile of TAP1 expression levels in multiple cancer types through ANOVA testing with an adjusted p-value of 0.05 (Fig. 1B). The data was extracted from TCGA, where we inquired in 33 tumor types paired with their normal samples for the mRNA expression of TAP1. We saw that among other cancer types, BRCA (breast cancer), LIHC (liver hepatocellular carcinoma), LUAD (lung adenocarcinoma), and OV (ovarian cancer) were significantly overexpressed. We also analyzed the TAP1 expression for different tumor tissues and their respective counterparts utilizing GENT2 through the HG-U133Plus2 database, and considered two sample t-test *p*-value of < 0.001 (Fig. 1C). The boxplot showed tissue wide expression of TAP1 among cancer experiments. The mRNA expression was significantly higher for breast, liver lung and ovary compared to the normal tissues.

### The pattern of TAP1 expression in breast, lung, liver, and ovarian cancer

**Fig. 2:**
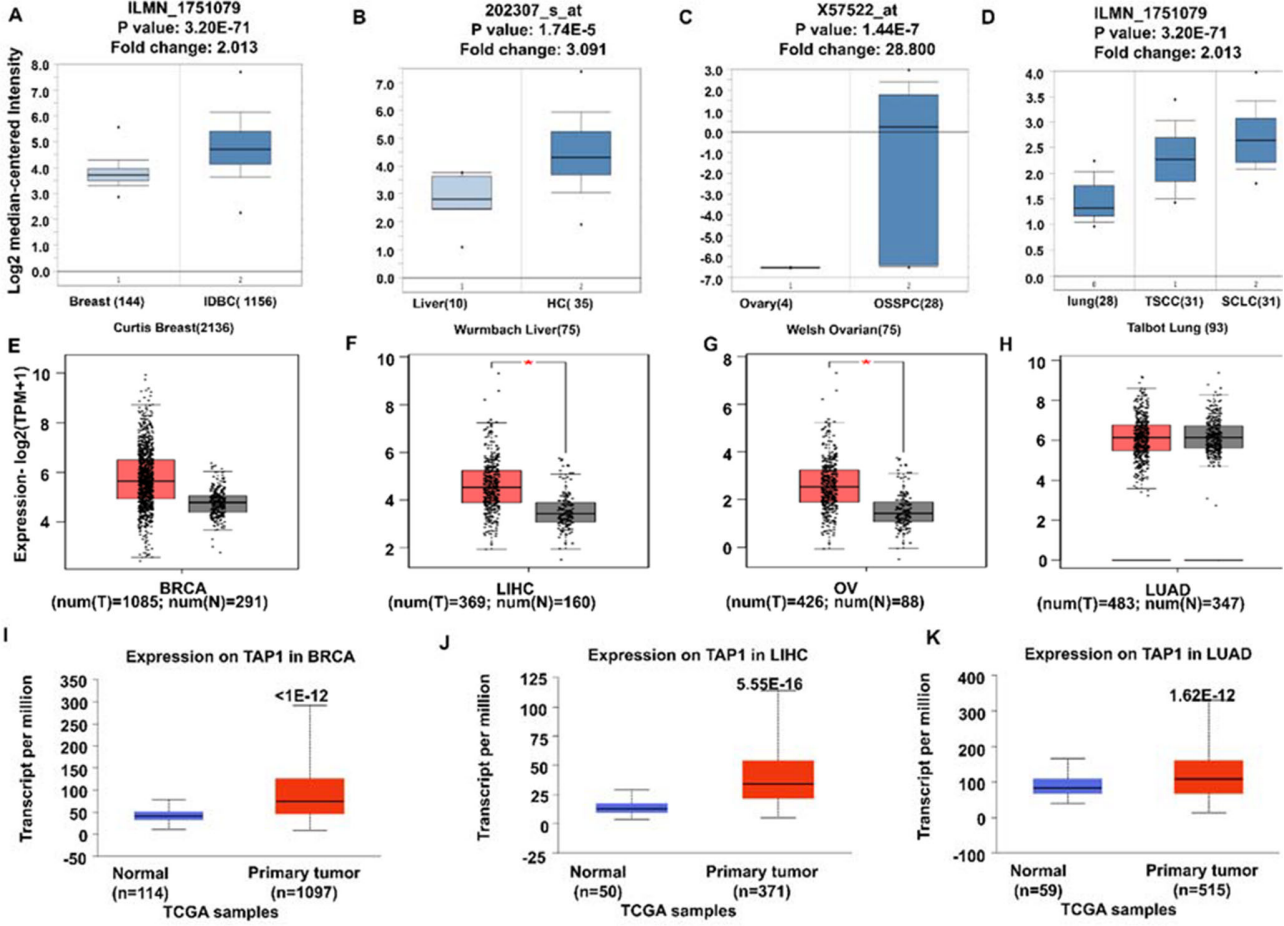
Cancer vs. normal comparison was observed in various cancers for the pattern of TAP1 expression. Box plot analysis for changes in the fold of TAP1 (log2 transformation of gene expression change) was conducted using four cancers namely: breast, liver, lung, and ovarian cancer where the left plot represents normal and right plot represents tumor cells, and the highest and lowest levels are shown by an asterisk (A–D) using the ONCOMINE analytical tool. (A) Invasive ductal breast carcinoma, (B) hepatocellular carcinoma, (C) ovarian serous surface papillary carcinoma, (D) tongue squamous cell carcinoma, squamous cell lung carcinoma. (E–H) The analytical expression pattern investigation of TAP1 was conducted by GEPIA2 utilizing ANOVA differential method. *p-value: 0.01. (I–K) were utilized by UALCAN web and TCGA samples were used to analyses the expression of TAP1 in primary tumor vs. normal cells

We further investigated the TAP1 gene expression pattern in various cancer kinds (Fig. 2). We used the ONCOMINE database to observe the gene expression and fold changes. It was proceeded by considering four cancer types: breast, lung, liver, and ovarian cancers. The expression was seen to be upregulated in breast, liver, ovary, and lung cancers (Fig. 2A–2D). The GEPIA2 tool was used to look into the expression of the TAP1 gene (Fig. 2E–2H), where the expression levels for LIHC and OV cancers were significantly higher than the normal tissues. The expression in the primary tumor and the normal was compared using the TCGA database in the UALCAN tool (Fig. 2I–2K). A significant overexpression for the TAP1 expression was seen in the primary tumor in comparison with the normal in BRCA, LIHC, and LUAD.

### The pattern of TAP1 protein expression in breast, lung, liver, and ovarian cancer

**Fig. 3:**
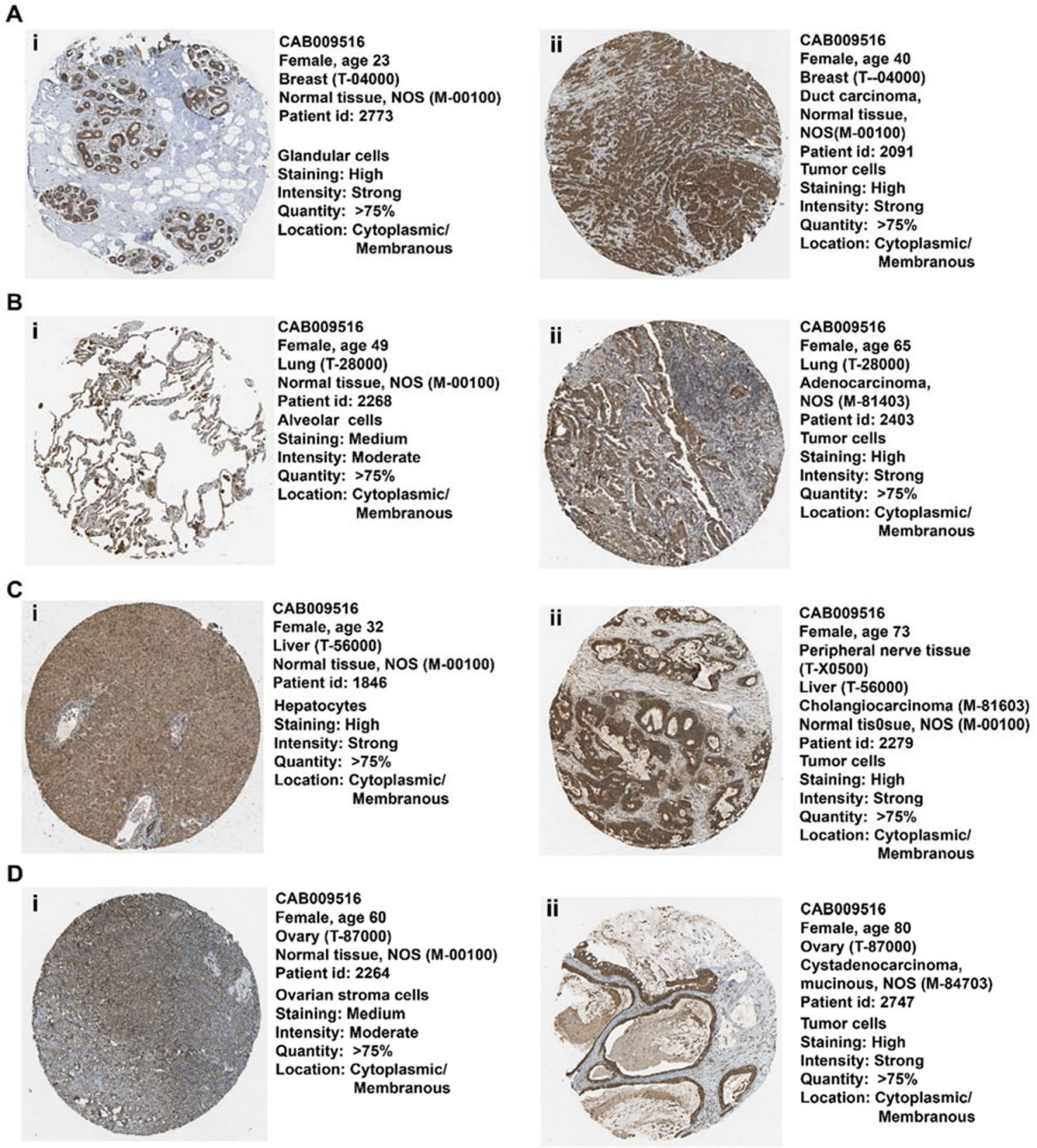
Expression of TAP1 protein in (A) breast duct carcinoma tissue compared to normal breast tissue. (B) Lung adenocarcinoma tissue compared to normal lung tissue. (C) Liver cholangiocarcinoma tissue to normal liver tissue. (D) Ovary cystadenocarcinoma tissue compared to normal ovarian tissue describing their immunochemistry using Human Protein Atlas database

We explored the expression of TAP1 protein derived from antibody-based protein profiling using immunochemistry for the different cancer tissues and their counterpart normal tissues (Fig. 3). The protein levels of TAP1 were high in all the cancer type tissues, with high staining and strong intensity (Fig. 3A (ii), 3B (ii), 3C (ii), and 3D (ii)). Though, we found high protein levels for granular breast and liver tissues as well (Fig. 3 A (i) and C (i)).

### Expression analysis of TAP1 gene with clinical characteristics

**Fig. 4:**
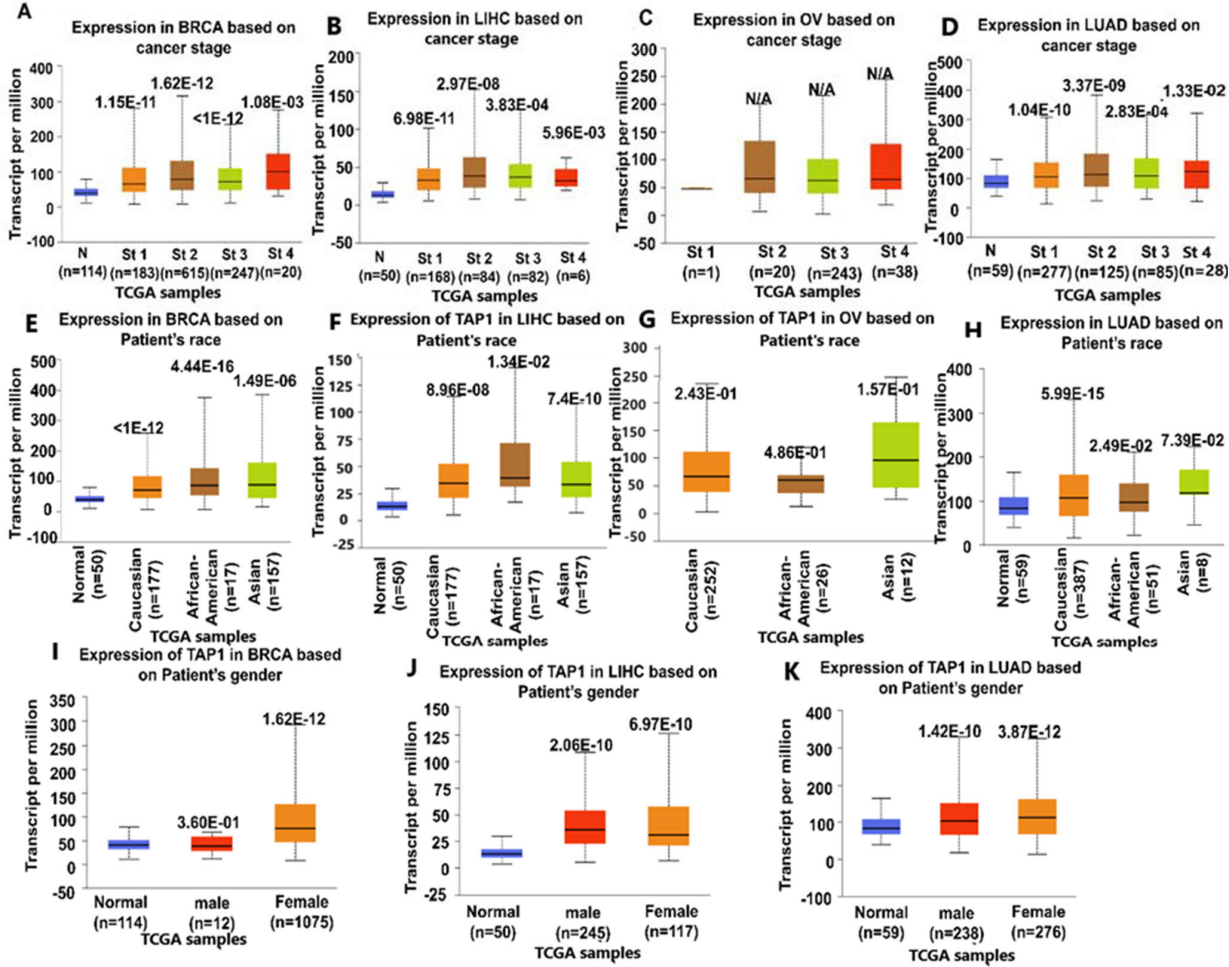
The relationship of the expression of TAP1 with different clinical characteristics in cancer affected people was shown in a box plot where the TAP1 mRNA expression level was detected through the UALCAN web. (A–D) Expression analysis for patient’s attributes based on specific cancer stages for BRCA, LIHC, OV, and LUAD, respectively. (E–H) The expression for the patient’s race for BRCA, LIHC, OV, and LUAD, and based on a patient’s gender (I–K) for BRCA, LIHC, and LUAD, respectively. Statistical significance assessed by the Student’s *t* test (unequal variance). *p < 0.05

We analyzed the expression of the TAP1 gene with different clinical characteristics using the UALCAN online database. The expression of the TAP1 gene in normal tissue was compared with tissues in patients with different clinical outcomes for breast, liver, lung, and ovarian cancers (Fig. 4 and Supplementary Table 1). Overexpression of TAP1 gene in cancer patient compared to normal tissue was most in stage 2 for LUAD (*p* = 3.37e-09) and LIHC (*p* = 2.97e-08) cancer, stage 2 (*p* = 1.62e-12) and stage 4 (*p* = 1.08e-03) were among the highest for BRCA. Stage 3 (p < 1e-12) had the least level of expression for TAP1 in BRCA, and stage 4 (*p* = 5.96e-03) showed the lowest level of expression for LIHC and stage 1 (*p* = 1.04e-10) and stage 4 (*p* = 1.33e-02) were among the lowest for LUAD (Fig. 4A, B, D). There was no significant TAP1 expression in ovarian cancer. All stages, for BRCA, LIHC, and LUAD, in comparison with the normal tissues had a significant elevated mRNA expression. Statistically significant overexpression of the TAP1 gene in cancer patients from different ethnicity compared to normal tissues were seen in BRCA, LIHC, and LUAD (Fig. 4E, F, H). TAP1 gene expression levels were significantly higher in females having breast cancer than normal counterparts (Fig. 4I, *p* = 1.62e-12). TAP1 expression had a significant difference for both, female vs normal, and male vs normal, for LIHC and LUAD (Fig. 4J, K).

### Promoter methylation of TAP1 gene with clinical characteristics

**Fig. 5:**
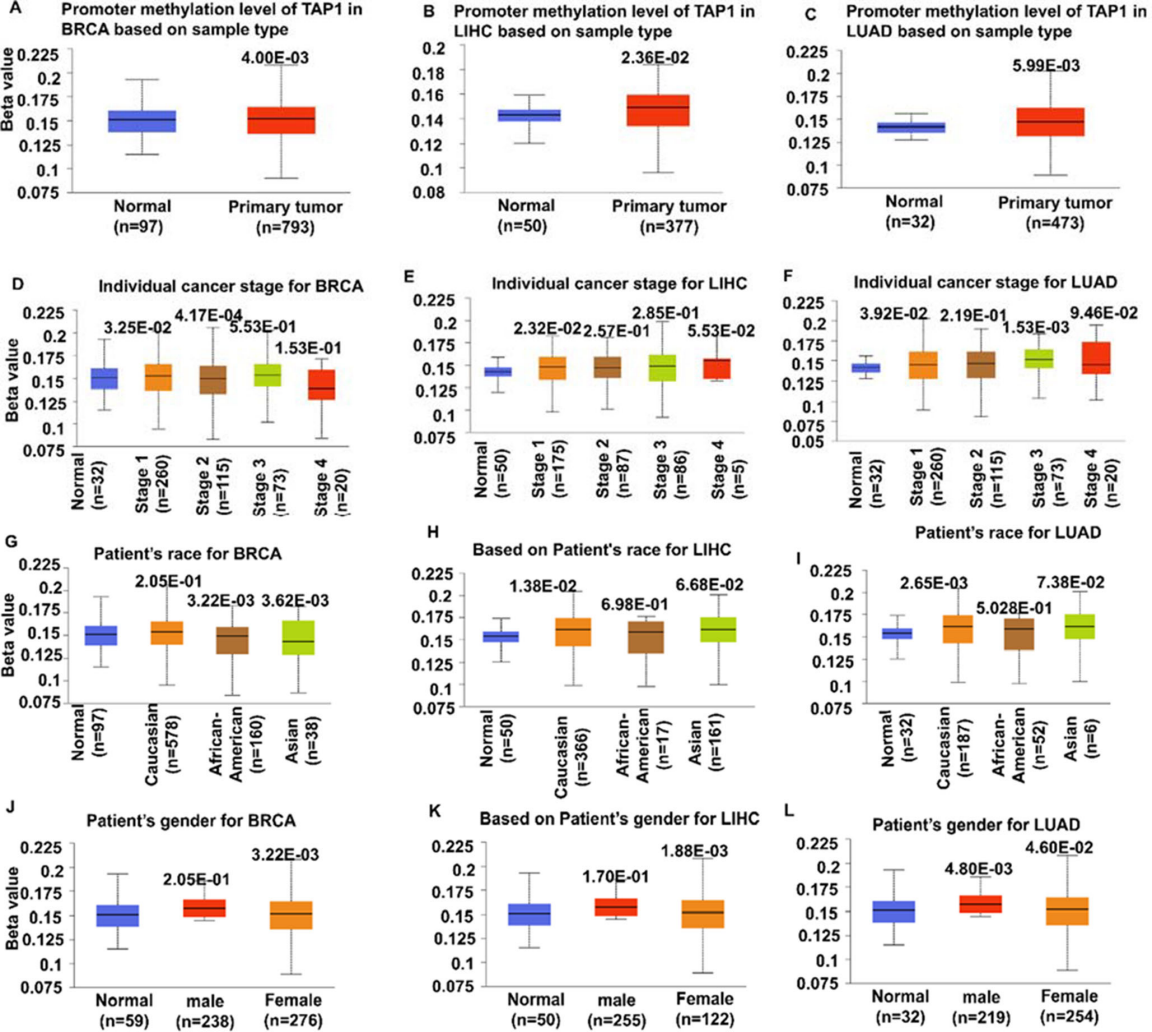
The relationship of the promoter methylation of TAP1 with different clinical characteristics in cancer affected people was shown in a box plot where the TAP1 mRNA promoter methylation level was detected through the UALCAN web. (A–C) The level of methylation of the primary tumor and in the counterparts in BRCA, LIHC, and LUAD, respectively. (D–F) Promoter methylation level for patient’s characteristics based on specific cancer stages for BRCA, LIHC, and LUAD, respectively. (G–I) Promoter methylation level for the patient’s race for BRCA, LIHC, and LUAD and based on a patient’s gender (J–L) for BRCA, LIHC, and LUAD, respectively. DNA methylation range: 0–1, 0, 0.5, and 1 respectively correspond to unmethylated, hemi-methylated (50% methylation), and fully methylated. Various cut offs ranging from 0.7 to 0.5 showing hypermethylation, 0.3–0.25 showing hypomethylation were taken into account

Note: There was no data available for OV in UALCAN for promoter methylation.

We analyzed the level of TAP1 gene promoter methylation with different clinical characteristics using the UALCAN online database. CpG probes were used to identify a correlation between the expression levels and promoter methylation. The box plots showed average beta value of DNA methylation in TCGA samples. The promoter methylation of the TAP1 gene in normal tissue was compared with tissues in patients with different clinical outcomes for breast, liver, lung, and ovarian cancer (Fig. 5 and Supplementary Table 2). Promoter methylation was increased significantly in tumor samples than normal tissues for BRCA (*p*= 4.00e-03), LIHC (*p* = 2.36e-02), and LUAD (*p* = 5.99e-03) (Fig. 5A–C). Compared to normal tissue, patients having stage 1 (*p* = 3.25e-02) and stage 2 (*p* = 4.17e-04) BRCA showed a significant upregulation in beta value (Fig. 5D), patients with LIHC had the highest promoter methylation in stage 1 (*p* = 2.32e-02) (Fig. 5E), patients having stage 1 and 3 LUAD had a significant elevation in promoter methylation than normal tissue (Fig. 5F). Compared to normal tissue, Asian and African American patients had increased promoter methylation in BRCA, and Caucasian patients had increased methylation for LIHC and LUAD (Fig. 5G–I). In breast, liver, and lung cancer, TAP1 promoter methylation was significantly unchanged for females compared to normal tissue (Fig. 5J–L). However, the analysis suggests no specific relation of TAP1 DNA methylation and clinico-pathological subtypes.

### Mutant mRNA, gene mutations, and copy number alterations of TAP1

**Fig. 6:**
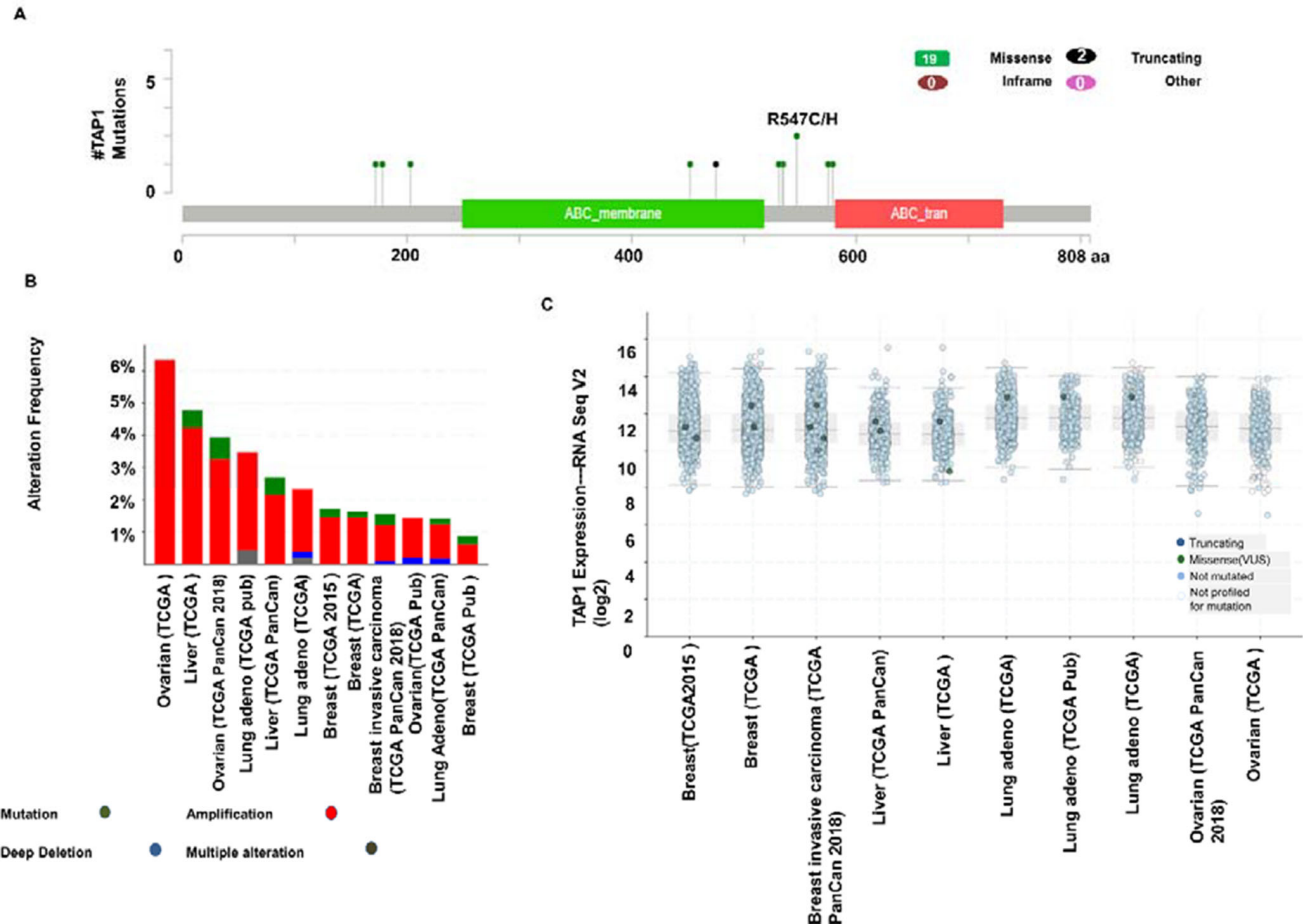
Analysis of expression of mutant mRNA, gene mutations, and copy number alterations of tap1 gene for different cancer studies using cBioPortal. (A) 21 Mutations were observed on samples between 1 and 808 amino acid residues in between pro-peptide and domain of TAP1 protein. (B) The frequency of alteration showing mutations (green), amplification (red), deep deletion (blue), and multiple alterations (gray) was presented graphically. Datasets having a minimum of 100 cases were plotted. (C) Truncating mutation (deep blue), a missense mutation (green), no mutation (light blue), and mutation not profiled (white) in TAP1 expression by RNA-Seq V2 method for a sample of 12 studies

By utilizing the cBioPortal database, genetic alteration of TAP1 in different cancers were studied. Generated database queried to observe the genetic mutation of TAP1 in 7710 specimens from 12 cases of BRCA, LIHC, LUAD and OV cancers. Of the total queried samples, there was a 2% alteration in the gene set or pathways with the somatic mutation frequency of 0.3%. Considering multiple sample studies, in total 21 mutations, including 9 duplications, were reported for the TAP1 gene area (Fig. 6A). We observed between 1 and 808 amino acids in the TAP1 pro-peptide and TAP1 domain for the query. Amidst those mutations, 19 were missense, and 2 truncating mutations were identified thoroughly. Breast adenocarcinoma and lung cancer had the highest level of mutations found in them, and the mutations laid among a hotspot in R547C/H. Two mutations were identified in the R547C/H. The site contained mutations, such as missense mutations, that were discovered in 8 breast adenocarcinoma specimens, 3 lung cancer expressed missense mutations. For ovarian cancer datasets, alteration frequency was found highest (>6%) among four cancer types (Fig. 6B). Consequently, it generated the expression of TAP1 mRNA (RNA Seq V2) among 12 cases of cancers by utilizing the cBioPortal (Fig. 6C). Breast cancer, 8 (7 missense and 1 truncating) cases, had the highest level of mutation in mRNA expression, and subsequently, the liver cancer was next in mutation with 4 affected.

### Prognostic investigation for the TAP1 expression among cancer patients

**Fig. 7:**
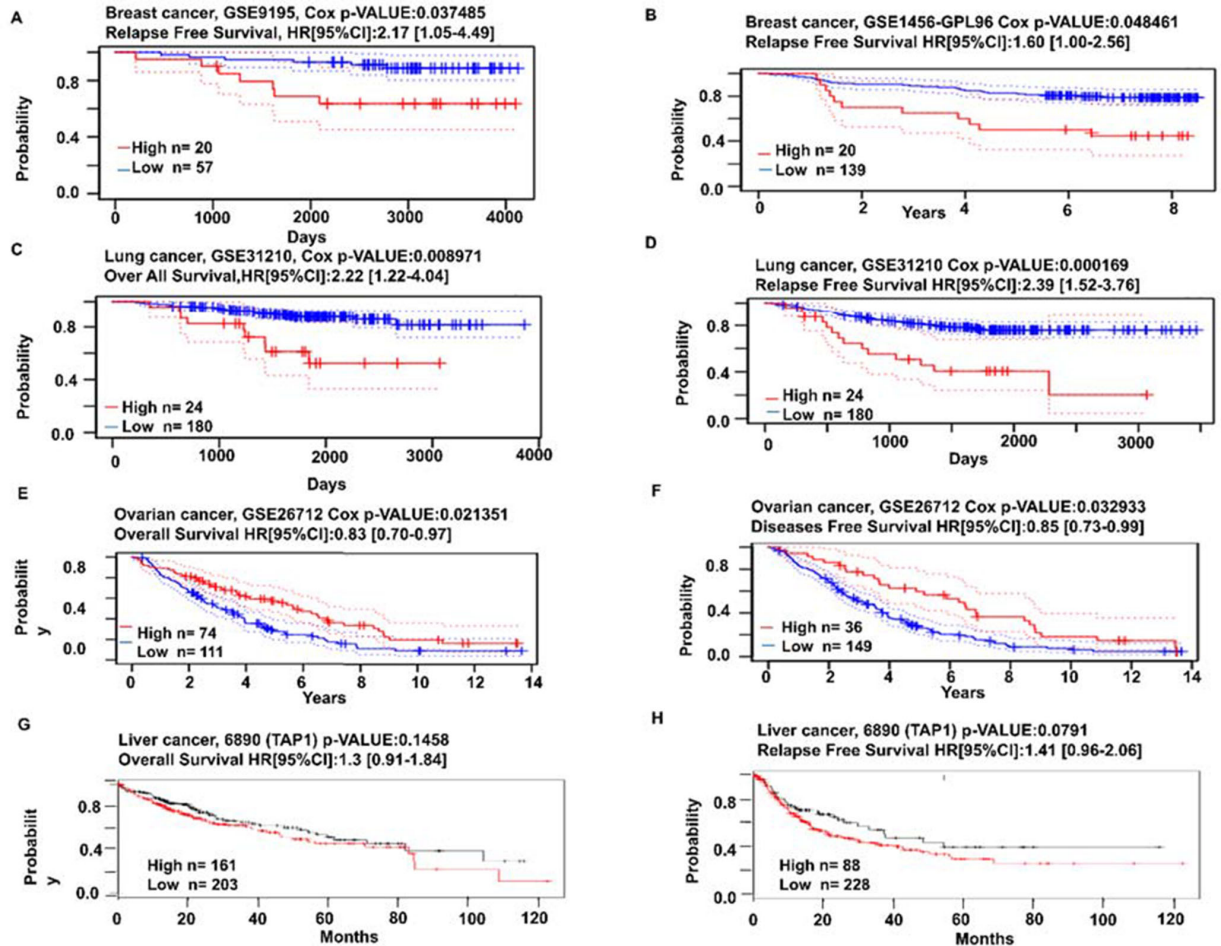
Analysis of patient survival on TAP1 expression in different cancers. The probability of affected people surviving with high (red) and low (blue) TAP1 expression. The plots were analyzed for cancers: (A, B) relapse-free survival in breast, (C, D) overall survival and relapse-free survival in lung, (E, F) overall survival and disease-free survival in ovarian, and (G, H) overall survival and relapse-free survival in the liver were retrieved from the PrognoScan Database, with cox *p*-value of 0.05. The probability of survival with high (red) and low (black) TAP1 expression curve concerning time in liver cancer (G, H) was retrieved from the KM plotter

Different categories of cancer were considered for the prognosis of TPA1 mRNA expression and we summarized the data using the prognostic databases with Cox p-value of a significance of (*p*<0.05). To analyze the interaction as for the expression of TAP1 with the ratio of survival for breast, lung, liver, and ovarian cancer patients, the PrognoScan database was used where the high and low TAP1 expressing groups were defined by log-rank test. In case of breast cancer, GSE9195 and GSE1456-GPL96 sets provided data that patients with decreased expression of TAP1 gene (*n* = 57 and *n* = 139 respectively) had significant higher relapse-free survival in comparison with the ones with a greater expression of TAP1 (*n* = 20, for both) (Fig. 7A, 7B). The results found for the lung cancer dataset had the same consequences as the breast cancer results, for OS and RFS (Fig. 7C, 7D). Here the GSE31210 and GSE31210 datasets of lung cancer exhibited that the low expression (*n* = 180, for both) of TAP1 mRNA exhibited a significantly higher OS in comparison with the higher TAP1 mRNA expressed (n= 24, for both cases) group. On the other hand, the analysis of dataset GSE26712 and GSE26712 of PrognoScan showed significantly lower OS and DFS of ovarian cancer, for the lower TAP1 mRNA expression (*n* = 111 and *n* = 149, respectively) in contrast with the higher levels of counterparts (*n*= 74 and *n* = 36, respectively) (Fig. 7E, 7F). For in lower TAP1 expression group (*n*= 203 and *n*= 228 respectively) on KM plotter for liver cancer high survival ratio was reported in case of OS and RFS, respectively, compared to the higher expression of the counterparts (*n* =161 and *n* = 88, respectively) (Fig. 7G, 7H). Primarily, the data-focused that indifferent to the single ovarian cancer difference in the expression, high TAP1 expression is in a positive correlation with the low survival rate in breast, lung, and liver cancers.

### Analyses of pathway and gene ontologies of co-expressed genes of TAP1

**Fig. 8:**
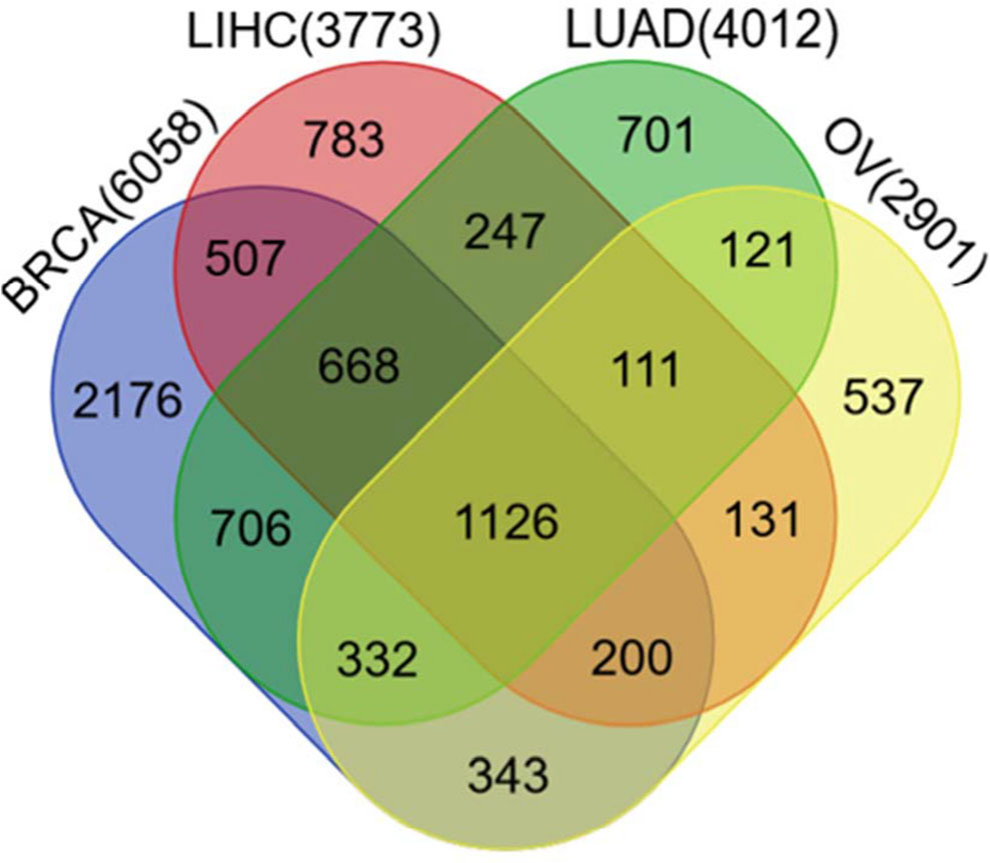
Venn diagram of positively correlated genes to the TAP1 gene for BRCA, LUAD, LIHC, and OV, collected from the Draw Venn web tool and R2 genomics web-based tool

Lastly, we figured out the genes that positively correlated with the TAP1 gene in BRCA, LIHC, LUAD, and OV cancer by using the R2 genomics analysis and visualization platform (Supplementary Table 3). The correlated genes were used in Venn Draw to draw a Venn diagram giving us the common correlated genes in BRCA (6058 genes), LIHC (3773 genes), LUAD (4012 genes), and OV cancer (2901 genes) (Fig. 8 and Supplementary Table 4).

**Fig. 9:**
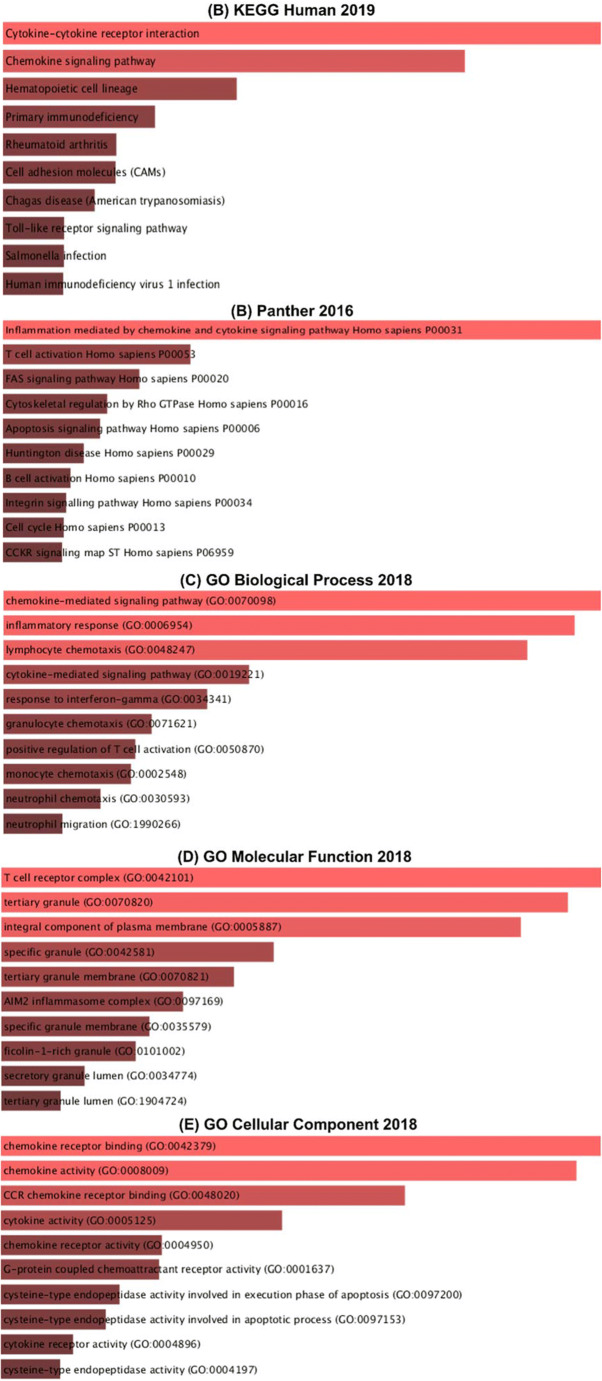
Signaling analysis of pathways and ontologies of genes with positive correlation with the TAP1 gene in BRCA, LUAD, LIHC, and OV was presented graphically using Enricher Web. (A) Kyoto Encyclopedia of Gene and Genomes (KEGG) Human pathways 2019, (B) Panther 2016, (C) Enrichment of GO Biological Process (2018) terms in proteomic analysis, (D) Enrichment of GO Molecular Function (2018) terms in proteomic analysis, and (E) Enrichment of GO Cellular Component (2018) terms in proteomic analysis. Generated bars, representing the ratio, % composition of terms in proteomic data:% composition of genomic annotation, are ranked by *p*-value

We extracted the positively correlated common genes to conduct an ontology investigation. We used the Enrichr software in order to understand which signaling pathways were influenced by the positively co-expressed genes and the TAP1 gene in BRCA, LIHC, LUAD, and OV. In the pathway analysis of the KEGG database, We saw that the most correlated 10 Kyoto Encyclopedia of Genes and Genomes (KEGG) pathway of TAP1 and the gene which are in positive correlation with TAP1, were primarily associated with cytokine-cytokine receptor pathway, chemokine signaling pathway, hematopoietic cell lineage, primary immunodeficiency, rheumatoid arthritis, cell adhesion molecules, Chagas disease, toll-like receptor signaling pathway, *Salmonella* infection, and human immunodeficiency virus-1 infection. Cytokine-cytokine receptor pathway and chemokine signaling pathway being the most significant pathways to be influenced (Fig. 9A). Panther database showed more significant interaction with inflammation mediated by chemokine and cytokine signaling pathways. It also showed pathways like T cell activation, FAS signaling pathway, cytoskeletal regulation by Rho GTPase, apoptosis signaling pathway, Huntington disease, B cell activation, integrin signaling pathway, cell cycle, and SSKR signaling map ST (Fig. 9B). The Enrichr tool also performed a gene ontology analysis to explore the different processes based on the co-expressed genes. The co-expression in biological processes was most significant for chemokine mediated signaling pathway (GO 0070098), inflammatory response, and lymphocyte chemotaxis (Fig. 9C). In molecular functions, the T cell receptor complex, tertiary granule, and integral component of the plasma membrane (Fig. 9D), and in GO cellular component, the chemokine receptor binding and chemokine activity were mostly influenced (Fig. 9E).

## Discussion

TAP protein complex portrays a crucial role in the MHC I pathway as it is responsible for delivering proteins to endoplasmic reticulum from cytosol, utilizing the energy harvested by ATP hydrolysis. The peptides are then displayed by MHC I in the infected cells so that it will be identified by the T cells [7]. Because of its role in tumor antigen presentation, it can be used as a target for cancer treatment [31]. The TAP protein constitutes of TAP1 and TAP2 heterodimers that are highly significant for the antigen presentation and a defect in one of the genes can conclude to an unstable TAP protein [32]. The heterodimers are regulated parallel. Furthermore, TAP1 stabilizes TAP2 to assemble TAP protein. So, TAP1 determines the amount of functional TAP protein-complex [33].

We have seen in previous studies that both the dimers are needed for a functional TAP protein complex. Alteration of TAP1, as shown in study, does not mutate TAP2 but a defect in TAP1 had an effect in the TAP2 expression. An indication that TAP1 is needed for a properly functional TAP2. An inadequate TAP protein cannot perform properly and lead to improper tumor antigen presentation and evasion of cancer immunity. Recently, many researchers worked on identification cancer biomarker using bioinformatics [35–38].

The tumor cells present tumor antigens, which are detected and killed by cytotoxic T cells. TAP1 gene codes, a transporter protein which being a component of MDR/TAP of ABC transporter family, has a vital role in chemotherapeutic drug resistance [39]. The TAP1 encoded protein is involved in the processing of antigens it contributes to anti-cancer drug resistance and hence, has an effect on the survival rates of patients and can play a part in cancer progression due to its MDR activity.

As tumor deaths depend on the transportation of proteins with the help of TAP1, it is important to know the expression analysis of TAP1 in the cancer cells. Though low TAP1 expression is related with tumor development and alteration of TAP1 may lead to evasion of cytotoxic T-cell killing of some cancer cells [40], but studies are also present about downregulating TAP1 as a way to promote neoantigens as tumor immunity [41]. The consideration of microenvironment for tumor seems essential regarding the expression of a gene as TAP1 as its regulation may be determined by proteins like STAT1 and IRF1. Therefore, resulting in varied expressions of the gene in different cancers [42]. We need analytical data to show the standpoint of TAP1 in various cancers. It is vital to have a systematic report for TAP1 in order to evaluate approaches towards future treatment. We, here, gather computational integrative omics data analysis for TAP1 in multiple cancers.

In previous studies, we have seen defective TAP protein resulting in an inadequate tumor antigen presentation, leading to tumor progression [43]. A decreased level of TAP1 was related to various cancers, like colon [44], lung [45], and cervical cancer [46]. A differential TAP1 expression has been linked to the liver [47] and renal cancer [42]. Studies also reported that a higher expression of TAP1 mRNA was found in stage II breast tumors and an increased level of expression was identified in high-grade breast cancers [48]. Interestingly, our results showed that TAP1 expressions were seen to be higher in cancer tissues in some cancers. In one of the papers [41], we saw the faulty allele of TAP1 was being associated with its transportation function in lung cancers. But in our study, we witness the expression of TAP1 to be higher in lung cancer tissues. The different analysis we performed helped us in understanding and to be surer of the result we got. We also wondered if TAP1 expression in different cancers were different, i.e. if certain cancers would have higher TAP1 result and another cancer kind will have a lower TAP1 expression hence, the analysis of expression in multiple cancers would help us be clearer on that aspect. To understand the role of TAP1 expression in different cancers, we used ONCOMINE, GEPIA2, and GENT2 databases. The expression pattern was studied among multiple cancer and their contrasting normal tissues, where the mRNA level was seen to be higher in breast, liver, lung, and ovarian cancers in GEPIA2 and GENT2. TAP1 was also overexpressed in ONCOMINE database for breast, liver and ovarian cancers but had a contradiction with other databases in the case of lung cancer. We found under expression of TAP1 for lung cancer in the ONCOMINE analysis, whereas GEPIA2 and GENT2 showed significant overexpression.

We focused on the expression of TAP1 in four cancers kinds, breast, liver, lung, and ovarian cancers. The analytical expression pattern in these cancers gave us a clearer idea about the regulation of the gene in each cancer and the fold change in the expression in cancer in comparison with the normal one in the ONCOMINE database. It provided us with data regarding the quantitative difference between normal and cancer cells, where all the cancers showed a noticeable fold change in the TAP1 expression. A significant rise in the TAP1 expression of LIHC and OV was seen in the ULCAN web. The TCGA database showed primary tumors to have a significant increase in TAP1 expression for BRCA, LIHC, and LUAD. For both the analysis, we found that LIHC had a higher expression. As reported earlier, TAP1 expression is increased multi-fold times in patients affected with the hepatitis C virus, which is a significant factor of liver cancer [49] [47]. Overall, we did find significant increase of TAP1 expression in cancer tissues in several databases, which indicates a relationship between TAP1 expression and multiple cancers.

To verify the trend we analyzed the expression of TAP1 protein levels in normal and cancer tissues of breast, liver, lung and ovaries by using the Human Protein Atlas project database. We found that the protein levels were high for all cancer types. The TAP1 protein levels were moderate in normal lung and ovary tissues and high in normal breast and liver tissues. We have seen high protein levels of both TAP1 and TAP2 in high grade breast cancer before [48]. It gave us more clarity as TAP1 protein levels were elevated for all four kinds of cancer.

Furthermore, we wanted to see if a patient with cancer had any clinical characteristics related to the TAP1 gene and how it varies in different cancers. We found that for BRCA, LIHC, and LUAD, all had a high expression of TAP1 gene for stage 2 carcinomas, indicating that that TAP1 might be present in an increased amount in stage 2 tumor in various cancers. The expression pattern fluctuates throughout the stages. In BRCA, it was low for the first stage and increased in the second, decreased substantially in stage 3, and increased again in stage 4. Both LIHC and LUAD had a similar pattern in terms of TAP1 mRNA expression. We reported an increase in expression for stages 2 and 3, and a decrease for stages 1 and 4. An explanation for such fluctuating results could be that the immune infiltrates are present in the cells, which induce interferon-gamma, and that induces TAP1 [48]. The IFN-y induction results when tumor cells escape targeting and INF-y induces TAP1 to trigger tumor detection as a part of the immune response. Even though, the TAP1 levels for different cancer stages showed fluctuating levels of TAP1 but the analysis showed significantly elevated TAP1 for BRCA, LIHC, and LUAD for all stages compared to the normal tissues. The univariant and multivariant study also showed a significant increase in females and different ethnicity with cancer in comparison their normal state for BRCA, LIHC, and LUAD.

Epigenetically changed DNA sequences contribute to gene expression and promoter methylation can downregulate the gene expression in cancers. In our data, there is a positive correlation between beta value and methylation status but a negative correlation with gene expression. The ABC transporters go through post-translational modifications [50] but there has been no significant evidence for post-translational regulation for TAP1 found [51]. It is expected that proteins in HLA/ MHC complex will be downregulated in tumors [52]. The promoters of the TAP1 gene were seen to be methylated in primary tumors, but the Stage 2 tumors tend to have a lower methylation rate, which acknowledges our result with expression patterns. Methylation in the promoter may conclude in inhibition of the gene transcription [53] and thus, contribute to the expression pattern according to this study. Altogether, we did not find correlation between methylation and expression analysis and further comprehensive exploration are needed to understand the molecular mechanisms.

Exploration of the copy numbers, types of mutations, and alteration of the genome will help understand the function of the TAP gene in cancer progression. A change such as these can result in altered gene expression [54]. Previously, we saw that lung cancer caused by impaired human leukocyte antigen (HLA)-1 complex had altered the TAP1 gene. The TAP1 gene influences the HLA-1 complex maturation. An altered TAP1 gene results in an ineffective HLA-1 complex and helps escape tumor detection by the immune cells [55, 56]. So, we looked into the mutations in the TAP1 domain using the cBioPortal database. Mutation sites were analyzed, and 21 mutations were found between 1 and 808 amino acids of TAP1 propeptide and TAP1 domain, in which 19 were missense and 2 were truncating. The mutation hotspot, R547C/H, was in between 500 and 600 amino acid residues. . How the mutations in the hotspot might affect the TAP function still needs to be explored. In query for the frequency of alteration, the highest frequency occurred in ovarian cancer with (>6%), and the highest mutant mRNA in RNA Seq V2 was found in breast cancer.

We wanted to do a systematic inquiry of the computational data to see if TAP1 affected the survival rate of patients with various cancers. In a previous study for colorectal cancer, we saw that there was correlation between TAP1 expression and prognosis in a positive way [40]. We investigated BRCA, LIHC, LUAD and OV cancers patients’ prognosis related to the TAP1 expression. The database PrognoScan, a microarray database, and km plotter were used to achieve the data for survival rate. Prognostic analysis on the BRCA, LIHC, and LUAD showed a negative correlation for TAP1 expression and overall, relapse-free and/or disease-free patient survival. However, for OV, the survival curve was higher for higher TAP1 expression because the analysis was conducted on patients with debulked tumors and debulking is related to increased survivals [57]. In a previous study, we saw that TAP1 was seen to be correlated with overall survival for high-graded serous ovarian cancer [58] but then again, we are considering just ovarian cancer in our study.

Giving us an expression that with an increased level of TAP1, there is a weaker probability of survival for the mentioned cancers. We analyzed the positively co-expressed genes with TAP1 and produced a Venn diagram using R2 genomics and Venn Draw to get the common correlated genes in BRCA, LIHC, LUAD, and OV. The identification of different pathways influenced by TAP1 and the common correlated genes can give us an idea about the mechanisms possibly being affected. An ontology analysis was performed using Enrichr. In KEGG pathway analysis, cytokine-cytokine receptor, antigen processing and presentation, chemokines seem to be most influenced. Cytokine receptors are involved in the initiation of JAK and signal transducer and activation of the transcriptions (STAT) pathway. JAK-STAT pathway stops the progression of tumor cells via tumor surveillance, but an excessive activation of this pathway was found in malignant tumors [59]. An inflammatory response caused by cancer results in a rise in chemokines [60]. Panther pathway showed a positive correlation with inflammation mediated by chemokine and cytokine signaling pathways. The GO pathways looked into the biological, cellular, and molecular pathways. Other than chemokines and inflammatory response, a significant interaction was with lymphocytes chemotaxis, which is responsible for migrating T cells into the tumor microenvironment [61].

In gene expression TAP is a heterodimer and may indicate that TAP1 protein stabilizes TAP2 protein, and both subunits are regulated in parallel

## Conclusion

This study demonstrated, for the first time, a thorough computational analysis of the TAP1 gene in multiple cancers to find a correlation between the TAP1 gene and tumor progression, using numerous recognized software and databases. We saw an upregulation of TAP1 mRNA not only in various cancers, but also, in primary tumors, different stages, ethnicities, and gender, when compared with normal tissues of BRCA, LIHC, and LUAD. Analysis in the databases revealed the mutation in TAP1, frequency alteration and copy number alterations of the gene and the pathways of correlated genes, which can be further studied to understand the mechanisms of TAP1 gene in cancer cells. We also reported a significant negative correlation for gene expression and the survival rate in the prognostic investigation in different cancer patients for BRCA, LIHC, and LUAD. The study has some interesting findings that can assist in further exploration of the TAP1 gene in oncogenic tissues, its role in cancer progression and tumor immune evasion.

## Supplementary information

ESM 1 (XLSX 12 kb)

ESM 2 (XLSX 12 kb)

ESM 3 (XLSX 841 kb)

ESM 4 (XLSX 11 kb)

## Data Availability

Not applicable.

## Abbreviations

ABC: ATP-binding cassette
ABCB: ATP binding cassette subfamily B member
BRCA: breast cancer
CNS: central nervous system
DFS: diseases-free survival
ER: endoplasmic reticulum
GTEx: genotype-tissue expression
HLA: human leukocyte antigen
IRF: interferon regulatory factor
LIHC: liver hepatocellular carcinoma
LUAD: lung adenocarcinoma
MHC-I: major histocompatibility complex
MDR: multidrug resistance
OV: ovarian cancer
OS: overall survival
RFS: relapse-free survival
STAT: signal transducer and activator of transcription
TCGA: the cancer genome atlas
TPM: transcript per million
TAP: transporter
TAP1: transporter associated with antigen processing 1
